# Ginsenosides on stem cells fate specification—a novel perspective

**DOI:** 10.3389/fcell.2023.1190266

**Published:** 2023-07-05

**Authors:** Ying Liu, Leilei Jiang, Wenbo Song, Chenxi Wang, Shiting Yu, Juhui Qiao, Xinran Wang, Chenrong Jin, Daqing Zhao, Xueyuan Bai, Peiguang Zhang, Siming Wang, Meichen Liu

**Affiliations:** ^1^ Northeast Asia Research Institute of Traditional Chinese Medicine, Changchun University of Chinese Medicine, Changchun, China; ^2^ Changchun Institute of Optics, Fine Mechanics and Physics, Chinese Academy of Sciences Changchun, Changchun, Jilin, China

**Keywords:** ginsenosides, stem cells, proliferation, fate specification, differentiation, self-renewal

## Abstract

Recent studies have demonstrated that stem cells have attracted much attention due to their special abilities of proliferation, differentiation and self-renewal, and are of great significance in regenerative medicine and anti-aging research. Hence, finding natural medicines that intervene the fate specification of stem cells has become a priority. Ginsenosides, the key components of natural botanical ginseng, have been extensively studied for versatile effects, such as regulating stem cells function and resisting aging. This review aims to summarize recent progression regarding the impact of ginsenosides on the behavior of adult stem cells, particularly from the perspective of proliferation, differentiation and self-renewal.

## 1 Introduction

*Panax ginseng* (*Panax ginseng* C. A. Mey.), is a perennial herb of the *Araliaceae* family (de Oliveira Zanuso et al., 2022). This plant is widely cultivated in East Asia, particularly in China, Japan, and South Korea, due to its characteristic of being both a food and a medicine (Ichim and de Boer, 2020). Ginseng contains ginsenosides, polysaccharides, proteins, polypeptides, amino acids and other chemical components, among which ginsenosides are the main medicinal components (Lee et al., 2019). Currently, around 200 types of ginsenosides have been reported (Ratan et al., 2021). Based on the classification of ginsenosides by glycoside type, ginsenosides generally be compartmentalized into two categories: dammarane-type tetracyclic triterpene and oleanane-type pentacyclic triterpene saponins (Hou et al., 2021). Dammarane-type ginsenosides are the primary types and biologically active components of ginsenosides, which are divided into protopanaxadiol (PPD) types (including ginsenosides Ra1, Ra2, Ra3, Rb1, Rb2, Rb3, Rc, Rd, Rg3, Rh2, F2, compound K, malonyl-Rb1, malonyl-Rb2, malonyl-Rc and malonyl-Rd, etc.) and protopanaxatriol (PPT) types (including ginsenosides Re, Rf, Rg1, Rg2, F1 and Rh1, etc.) (Pan et al., 2018). In contrast, oleanane-type ginsenosides (including Ro, Rh3, Ri, etc.) are rare in ginseng species (Zhang H. et al., 2022). The experimental pharmacological research of ginsenosides have shown that the number of sugar residues contained in the branched, the position of glycosides, and their stereoselectivity all affect the pharmacological activity of ginsenoside monomers (Piyasirananda et al., 2021; Yousof Ali et al., 2021; Ali et al., 2022). Oral administration of ginsenosides is the major approach, but they are not easily absorbed by themselves with low bioavailability (Won et al., 2019). On the contrary, a large number of enzymes or gut microbiota can convert ginsenosides into deglycosylated products with a higher bioavailability and pharmacological activity that can be easily absorbed by human body (Yang L. et al., 2020). Ginsenosides have been found to possess a range of pharmacological effects, such as anti-aging (de Oliveira Zanuso et al., 2022), anti-tumor (Wong et al., 2015), hematopoietic recovery (He et al., 2021), promotion of osteogenesis (Wu et al., 2022), and neuroprotection (Rokot et al., 2016). In clinical trials, ginsenosides have been beneficial to the treatment of acute ischemic stroke, cancer, and chronic kidney disease, and can prompt oxidative stress or inflammation caused by exercise challenges (Table 1). Although ginsenosides exhibit various positive physiological activities, this review will focus on the relationship between ginsenosides and stem cells.

**TABLE 1:** Clinical efficacy of ginsenosides.

Component	Application	Effect	Mechanism	References
Rd	AIS	neuroprotection	microglial proteasome activity and sequential inflammation↓	Zhang et al. (2016)
Rg3	AL	anti-angiogenic	PI3K/Akt and ERK1/2 pathways↓	Zeng et al. (2014)
Rb1	CKD	alleviate kidney dysfunction	oxidative stress and inflammation↓	Xu et al. (2017)
Rg1	sports challenge	reduce oxidative damage and inflammation	TBA activity↓ and TNF-α mRNA↓ and IL-10 mRNA↑	Hou et al. (2015)
Rg1	exercise resistance	induces immune stimulation and reduces skeletal muscle aging	p16^INK4a^ and MPO mRNA levels↓	Lee et al. (2021)

AIS, acute ischemic stroke; AL, acute leukemia; CKD, chronic kidney disease.

Stem cells, encompassing both adult stem cells and embryonic varieties, are essential for developing human tissues and maintaining homeostasis (Zakrzewski et al., 2019). Their unique properties of self-renewal, high proliferation, and differentiation into multiple lineages make them attractive for a variety of applications, such as cell replacement as well as tissue and organ renewal in regenerative medicine (Brassard and Lutolf, 2019), exploration of regulatory mechanisms during embryonic development (Weatherbee et al., 2021), therapy for various diseases (Sinenko et al., 2021), the establishment of disease models (Sterneckert et al., 2014) and drug screening and development (Kumar et al., 2021). Notably, the fate specification of stem cells (proliferation, differentiation and self-renewal) is involved in the regulation of the body’s biological process. An imbalance in fate specification can lead to the emergence and progression of aging or even disease (Chandel et al., 2016). Excessive proliferation of stem cells can lead to the development of tumors and cancers (Najafi et al., 2019), while impaired self-renewal and differentiation of stem cells can limit the potential of tissue and organ regeneration and damage repair, such as in the case of aging and nervous system injury (De et al., 2021; Sivandzade and Cucullo, 2021). Consequently, understanding the regulation of stem cells fate is indispensable for the prevention of aging and many diseases. The stem cell niche refers to the microenvironment that maintains the proliferation, differentiation and self-renewal of stem cells (Hicks and Pyle, 2023). Stem cells can receive signals from the ecological niche and respond accordingly, which plays an important role in supporting and coordinating the activities of stem cells (Chacón-Martínez et al., 2018). Ginsenosides inhibit inflammatory responses and reduce oxidative stress to improve the stem cells niche (Hu et al., 2015; Wu et al., 2020). Also, ginsenosides can promote stem cell proliferation, differentiation into specific cell types, or self-renewal, thus regulating stem cell function (He et al., 2019; He and Yao, 2021). In this review, we summarize the effects of various ginsenosides on adult stem cells, especially mesenchymal stem cells (MSCs), hematopoietic stem cells (HSCs), neural stem cells (NSCs) and cancer stem cells (CSCs), thereby elucidating the underlying mechanisms of ginsenosides in regulating fate specification of stem cells.

## 2 Transport and metabolism in stem cell niche of ginsenosides

The stem cell niche refers to the microenvironment at a specific location in a tissue or organ, which provides the necessary support and regulation for stem cells to maintain their proliferation, differentiation and self-renewal capabilities (Chacón-Martínez et al., 2018). The niche consists of stromal cells and the factors they secrete, such as adhesion molecules, soluble factors (cytokines, growth factors, metabolites, and nutrients), and matrix proteins. In addition, physical factors such as calcium ions and oxygen concentration also influence the characteristics of the stem cell niche. Recently the transport and metabolism of prototypical ginsenosides or ginsenoside metabolites in various types of stem cell niches have received extensive attention. ATP-binding cassette (ABC) transporters (especially ABCB1 and ABCG2) are clinically important transporters and drug efflux pumps, and their expression affects the differentiation activities of NSC stem cells (Lin et al., 2006). Specifically, downregulation of ABCB1 (also known as P-glycoprotein, p-gp) or ABCG2 (BCRP) expression promotes the differentiation of NSCs into astrocytes or neurons (Lin et al., 2006). Ginsenosides and their metabolites (CK, PPD, and PPT) have been studied to be potential inhibitors of p-gp and BCRP (Jin et al., 2006; Li et al., 2014). Those findings suggest that ginsenoside metabolites may antagonize ABC transporter expression, thereby benefiting NSC differentiation. Furthermore, overexpression of ABC transporters in CSC supports drug resistance (Li et al., 2010). The inhibition of ginsenosides on the efflux effect of ABC transporters may be one of the means to promote the sensitivity of CSCs to chemotherapeutic drugs. In addition, the enzymes involved in drug metabolism are mainly cytochrome P450 (CYP450), including CYP2C9, CYP3A4, etc. The inhibition of CYP450 prolongs the metabolism time of the drug in the body and increases the blood drug concentration. CYP450 is highly expressed in the bone marrow niche (HSC living environment) (Zhang Y. et al., 2013). The competitive inhibition of ginsenoside metabolites (CK, PPD, and PPT) on the activity of liver drug enzymes (CYP2C9, CYP3A4) may be the reason why they reside in the bone marrow niche and exert their pharmacological effects, thereby regulating HSC function (Liu et al., 2006). Recently, the transport of ginsenosides in the NSC niche was found that the active transport of ginsenoside Rb1 to brain microvascular endothelial cells, the cellular component of the NSC niche, was dependent on the glucose transporter GLUT1 (Wang et al., 2018). This finding suggests that upregulation of GLUT1 can increase the bioavailability of ginsenosides in NSCs and their niche. In the future, more *in vivo* experiments are needed to screen and verify the key enzymes/proteins related to the transport and metabolism of ginsenosides in stem cells and niches, which will help the uptake of ginsenosides by stem cells.

## 3 Effects of ginseng on different types of stem cells

Stem cells are pluripotent cells with the capacity for self-renewal, self-replication, and differentiation into multiple cell types in a suitable microenvironment, which are divided into two forms according to developmental stages: embryonic stem cells and adult stem cells. Considering that embryonic stem cells are subject to ethical restrictions, are prone to self-differentiation, and may have abnormal karyotypes after many passages, the research on the effect of ginsenosides on stem cells mainly focuses on adult stem cells (Scott and Reijo Pera, 2008). Adult stem cells, including hematopoietic, neural, and mesenchymal stem cells, are slow-dividing and quiescent cells with low proliferative rates and are the source of adult tissue (Gurusamy et al., 2018). Additionally, adult tissue stem cells can turn into cancer stem cells (White and Lowry, 2015), in solid tumors, the acquisition of cancer stem cells phenotype can be achieved through epithelial-mesenchymal transition (EMT) (Shibue and Weinberg, 2017). The wide application of stem cells in regenerative medicine has attracted much attention. At first, stem cells were transplanted into the human body to repair damaged tissues, utilizing their potential for self-replication and multi-directional differentiation (Giri et al., 2019). Furthermore, advancements in stem cells reprogramming technology have led to increased use of stem cells for the restoration of aging cells, that is, stem cells can restore proliferate, differentiate, and self-renewal ability to delay the aging process (Alle et al., 2021). Recently, stem cells were suggested as a promising therapeutic option for various diseases, including but not limited to neurodegenerative diseases, cancer, stroke, myocardial ischemia (Yamashita and Abe, 2016; Michler, 2018; Sivandzade and Cucullo, 2021; Yin et al., 2021). Meanwhile, ginsenosides have been proven to slow the pathological process of these diseases and improve the condition (Huang et al., 2019; Wang R. et al., 2020; Yang JE. et al., 2020; Yao and Guan, 2022). In recent times, research studies have demonstrated the significant regulatory impact of ginsenosides on the self-renewal, differentiation, and proliferation of stem cells, thereby highlighting their potential for clinical use in improving the field of stem cells research (Table 2).

**TABLE 2:** Changes and effects of ginsenosides on the physiological behavior of various stem cells.

Cell type	Derive	Saponins	Effect	Targets/pathways	References
CSCs	colon	20(R)-Rg3	stemness and EMT↓	SNAIL signal axis↓	Phi et al. (2019b)
	colon	Rd	stemness and EMT↓	EGFR signal axis↓	Phi et al. (2019a)
	colon	CK	stemness and cancer metastasis↓	Nur77-Akt feedforward signaling↓	Zhang et al. (2022b)
	lung	Rk1/Rg5	EMT↓	Smad and NF-κB/ERK↓	Kim et al. (2021)
	breast	Rg3	stemness and self-renewal↓	Akt mediated self-renewal↓	Oh et al. (2019)
	skin/liver	Rh2	cell growth↓	Autophagy↑; β-catenin↓	Liu et al. (2015b), Yang et al. (2016)
	ovarian	Rb1	self-renewal↓	Wnt/β-catenin↓	Deng et al. (2017)
LSCs	CD34(+) CD38(−) LSCs	Rg1	proliferation↓ and cellular senescence↑	SIRT1/TSC2↑;p16INK4a↑ and hTERT↓	Tang et al. (2020a), Tang et al. (2021)
MSCs	human adipose	Rg1	proliferation↑ and adipogenic differentiation↑	Adipocytokine↑, IL-17↓	Xu et al. (2022)
	bone marrow	Rg1	aging↓	NRF2 and Akt↑; GSK-3β phosphorylation ↓ and Wnt↓	Wang et al. (2020b), Wang et al. (2021)
	bone marrow	Rg1	osteogenic differentiation↑	GR/BMP-2↑	Gu et al. (2016)
	bone marrow	Rg1	oxidative stress-induced apoptosis↓	PI3K/Akt↑	Hu et al. (2016)
	bone marrow	Rb1	migration↑	SDF-1/CXCR4 axis and PI3K/Akt↑	Liu et al. (2022b)
	bone marrow	20(S)-Rb2	Dex-induced apoptosis↓	GPR120↑, Ras-ERK1/2↑	Gao et al. (2015)
	human umbilical cord	Rg1	proliferation↑ and differentiation to NSCs↑	Wnt/β-catenin↓ and Notch↓	Xiao et al. (2022)
	muscle	Rb1	oxidative stress and mitochondrial dysfunction↓	NF-κB↓	Dong et al. (2022)
HSCs	Sca-1(+) HSC/HPCs	Rg1	HSCs aging↓	SIRT6↑, NF-κB↓; SIRT1-FOXO3 and SIRT3-SOD2↑; oxidative stress↓ and Wnt/β-catenin↑; p16(INK4a)-Rb and p19(Arf)-p53-p21(Cip/Waf1)↓; p53-p21-Rb signal↓	Chen et al. (2014), Yue et al. (2014), Tang et al. (2015), Li et al. (2016), Cai et al. (2018), Tang et al. (2020b), Zhou et al. (2020b), Wang et al. (2022a)
NSCs	-	Rg1	aging↓	Wnt/β-catenin↓; Akt/mTOR↓	Chen et al. (2018), Xiang et al. (2019)
	-	20(S)-PPD	proliferation↓ and differentiation↑	cell cycle↓, autophagy↑	Chen et al. (2020)
	endogenous	CK	neurogenesis↑	LXRα↑	Zhou et al. (2020a)

CK, Compound K.

### 3.1 Mesenchymal stem cells

Mesenchymal stem cells (MSCs) are the progenitors of numerous cell types and have the capacity to proliferate and differentiate into a variety of cell lineages such as osteoblasts, adipocytes, myoblasts, and others (Xie et al., 2020).

#### 3.1.1 Effect on differentiation of MSCs

Ginsenosides have shown the potential to induce differentiation of MSCs *in vitro*, especially in inducing osteogenic differentiation, which is the consequence of expressing or activating genes/transcription factors and signaling pathways related to osteogenic differentiation (He et al., 2019). The role of BMP-2/Smad pathway in MSCs osteogenic differentiation has been fully confirmed (Aquino-Martínez et al., 2017). As an indispensable growth factor for driving osteogenic differentiation, BMP-2 can activate the intracellular Smad pathway to form Smad complexes that enter the nucleus and promote the expression of the osteogenic transcription factor RUNX2 (Wang et al., 2017). Such the BMP-2/Smad signaling pathway can be activated by ginsenoside Rg1 to promote osteogenic differentiation of bone marrow mesenchymal stem cells (BMSCs), which is mediated by glucocorticoid receptor (GR) nuclear translocation (Gu et al., 2016). In addition, the Wnt/β-catenin signaling pathway is a key pathway that regulates the osteogenic differentiation of MSCs by regulating the localization of β-catenin, thereby regulating the expression of downstream osteogenesis-related proteins and genes. GSK-3β is an intermediary of the Wnt/β-catenin signaling pathway, and its high activity negatively affects the stability and transcriptional activity of β-catenin, blocks the activation state of the signaling pathway, and thus regulates the function and fate of stem cells. Currently, researchers believe that ginsenosides regulate the osteogenic differentiation of MSCs by regulating the Wnt signaling pathway. For instance, ginsenoside Rg1 can regulate the differentiation capacity of MSCs, stimulate bone and cartilage formation, by inhibiting the phosphorylation of GSK-3β and reducing the excessive activation of the Wnt/β-catenin pathway in aging cells (Wang Z. et al., 2020). Conversely, ginsenoside compound K (CK) (the main metabolite of original propanediol ginsenoside in gut bacteria) activates the Wnt/β-catenin signaling pathway *in vitro* and promotes the expression of the downstream Wnt target gene Runx2 (osteogenic transcription factor), inducing the osteogenic differentiation of rat bone marrow-derived mesenchymal stem cells (rBMSCs) (Ding et al., 2022). In view of the fact that ginsenosides have two sides to the regulation of Wnt/β-catenin signaling pathway in promoting osteogenic differentiation of MSCs, that is, they show differences in different states (aging or normal) of MSCs. Future detailed functional exploration of individual members of the Wnt/β-catenin pathway will help to understand its regulatory mechanism.

Adipose tissue-derived MSCs (ADSCs) are also quite common in clinical practice. Ginsenoside Rg1-promoted cartilage gene expression in ADSCs *in vitro* induces cartilage phenotype differentiation (Xu et al., 2015; Guo et al., 2023). Co-administration of ginsenoside Rg1 and platelet-rich fibrin elevates cytokines (VEGF, HIF-1α) in human ADSCs niche and promotes soft tissue regeneration (Xu et al., 2016). Ginsenoside Rg1 can also improve the ADSC niche mediated by adipokine and IL-17 signaling pathways, and promote the adipogenic differentiation of human ADSCs (Xu et al., 2022). These results indicate that ginsenoside may expand MSCs by regulating MSC niche.

#### 3.1.2 Effect on the proliferation and differentiation of MSCs in the aging process

**FIGURE 1:**
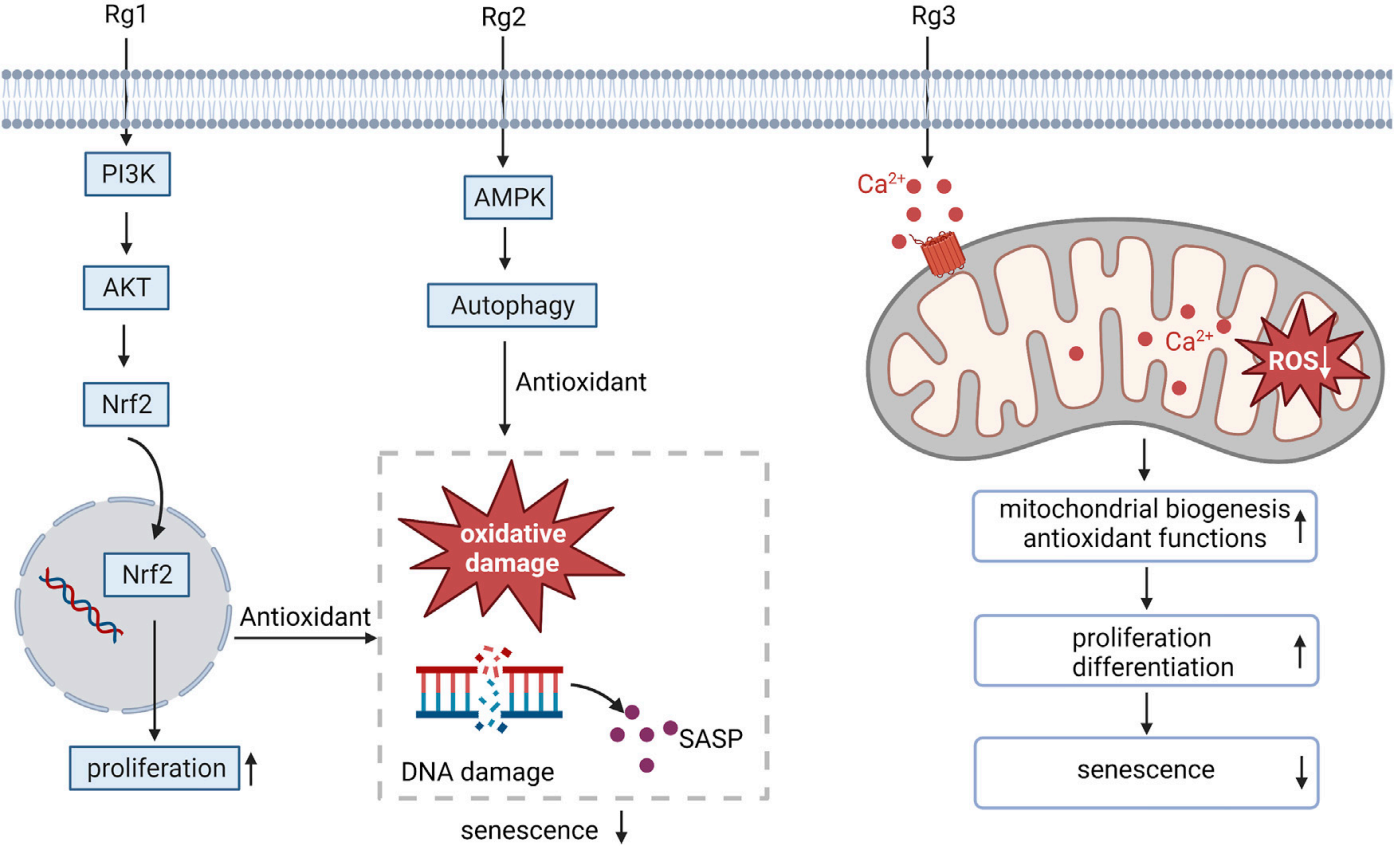
Ginsenosides resist oxidative stress, regulate MSC proliferation and differentiation, and alleviate MSC aging. SASP, senescence-associated secretory phenotype.

Oxidative stress is the key contributor to the aging of stem cells (Chen et al., 2017). Ginsenoside Rg1 has been proven to promote superior antioxidant and anti-inflammatory capabilities, which can promote BMSC proliferation and improve the anti-aging hematopoietic microenvironment (Hu et al., 2015). Similarly, the senescence-associated secretory phenotype (SASP) resulting from DNA damage and oxidative damage associated with the aging of MSCs can be inhibited by ginsenoside Rg1, which promotes MSC proliferation by enhancing its antioxidant capacity, the activation of Nrf2 and PI3K/Akt is required for this process to occur (Wang et al., 2021). Ginsenoside Rg2 activates AMPK-mediated autophagy restores pig MSCs proliferation and inhibits oxidative stress-induced replicative senescence in pig MSCs (Che et al., 2023). Ginsenoside Rg3 mainly enhances the biogenesis ability of mitochondria and antioxidant function by promoting Ca^2+^ concentration properly, thus improving proliferation and differentiation potential and preventing human MSCs from aging (Hong et al., 2020). These results suggest that administration of ginsenosides may be a promising approach to counteract MSC aging by intervening in oxidative stress-related pathways (Figure 1).

### 3.2 Neutral stem cells

During central nervous system (CNS) development, neural stem cells (NSCs) are capable of generating neurons, astrocytes, and oligodendrocytes (Vieira et al., 2018). Recently, the use of neural stem cells therapy has emerged as a novel approach to treating diverse neurological disorders and is an ideal approach for treating neurodegenerative diseases and CNS injuries (Liu Y et al., 2020).

#### 3.2.1 Effect on proliferation and differentiation of NSCs

Ginsenoside Rg1 increases the activity of NSE, a neuron biomarker, in transplanted NSCs, implying neuron-like differentiation (Li et al., 2015). Similarly, the key to ginsenoside Rb1 regulating the progression of neurodegenerative diseases is to increase the levels of biomarkers such as Nestin (marking NSC), GFAP (marking astrocytes) and NSE in Alzheimer’s disease (AD) rat models, and promote NSC proliferation and differentiation into astrocytes and neurons (Zhao et al., 2018). However, high levels of NSE have been shown to induce nerve injury and neuroblastoma, and the specific mechanism of how ginsenoside promotes NSE expression to exert neuroprotective effects needs to be further elucidated. Even though most ginsenosides are difficult to penetrate the blood-brain barrier due to their large molecular weight, their neuroprotective effects have been confirmed by a large number of experimental studies (Xie et al., 2018). The underlying mechanism may involve in improving the niche of NSCs. Nerve growth factor (NGF), a neurotrophic factor in the NSCs niche, can induce the differentiation of NSCs derived from the brain (Regalado-Santiago et al., 2016). Ginsenoside Rg1 precisely acts as an analog of NGF, attenuating oxygen and glucose deprivation-induced nerve injury and promoting proliferation and glial-like differentiation of cortical NSCs (Gao et al., 2017).

#### 3.2.2 Effect on proliferation and differentiation of NSCs in the aging process

Recently, the function of WNT/β-catenin in the CNS has been studied and dysregulation of its signaling can lead to the production and aggregation of β-amyloid (Aβ) (Aghaizu et al., 2020). Ginseng total saponins extract and its intestinal metabolite 20(S)-protopanaxadiol (PPD) jointly induce the phosphorylation of GSK-3β (ser9), activate the Wnt/GSK-3β/β-catenin pathway to promote NSC proliferation and differentiation, thereby improving cognitive impairment in AD by replacing damaged neurons (Lin et al., 2020; Lin et al., 2022). However, in LiCl-induced NSC senescence, ginsenoside Rg1-dependent downregulation of phosphorylated GSK-3β expression interfered with the activation of the Wnt/β-catenin pathway, thereby promoting NSC proliferation and delaying senescence (Xiang et al., 2019). Screening potential Wnt/GSK-3β/β-catenin -targeted activators/inhibitors in ginsenosides will help ginsenosides promote the development of stem cell regenerative medicine and anti-aging drugs in nerves field.

Aging leads to a decline in the capacity of NSCs to enter the cell cycle efficiently (Audesse and Webb, 2020). Genes involved in cell cycle regulation play an essential role in the stability and activation of NSCs (Roccio et al., 2013). Ginsenoside Rg1 targets Akt/mTOR to downregulate the levels of cell cycle arrest-related proteins (p53, p16, p21, and Rb) in NSCs, promoting NSC proliferation and alleviating D-galactose-induced NSC aging (Chen et al., 2018). 20(S)-PPD induces autophagy and cell cycle arrest, and promote NSC from a proliferative state to a differentiated state and helps to repair neurons in age-related neurodegenerative AD (Chen et al., 2020). This suggests that further understanding of the molecular mechanism by which ginsenosides alleviate NSC aging requires a deeper study of upstream signaling pathways and regulators that affect the cell cycle to maintain the continued health of NSCs.

#### 3.2.3 Effect on transdifferentiation of NSCs

NSCs can also differentiate from other stem cells with transdifferentiation capability, such as MSCs (Feng et al., 2014). It was previously demonstrated that ginsenoside Rg1 promotes the differentiation of transplanted bone marrow mesenchymal stem cells (BMSCs) into neurons and glial cells (Bao et al., 2015). A recent report has shown that ginsenoside Rg1 regulates miRNA-124 expression *in vitro* to promote neural differentiation of mouse adipose stem cells (ADSCs) (Dong et al., 2017). Ginsenoside Rg1 promotes the neural phenotype differentiation of human ADSCs by activating the expression of NSC niche components including growth associated protein-43 (GAP-43), neural cell adhesion molecule (NCAM), and synapsin-1 (SYN-1) (Xu et al., 2014). In addition, ginsenoside Rg1 promotes the differentiation of human umbilical cord mesenchymal stem cells (hUCMSC) into NSCs by downregulating genes involved in the Wnt/β-catenin and Notch signaling pathways, including GSK3β, β-catenin, Notch1, and Hes1 (Xiao et al., 2022). However, the transdifferentiation capability of NSCs is still doubted since the possible contamination by other tissue stem cells or embryonic stem cells in those studies.

### 3.3 Hematopoietic stem cells

HSCs have self-renewal potential and can differentiate into various hematopoietic progenitor cells (HPC) and produce specific blood cell types to maintain the stability of the entire hematopoietic system (Zhao and Li, 2015).

#### 3.3.1 Effect on proliferation of HSCs

**FIGURE 2:**
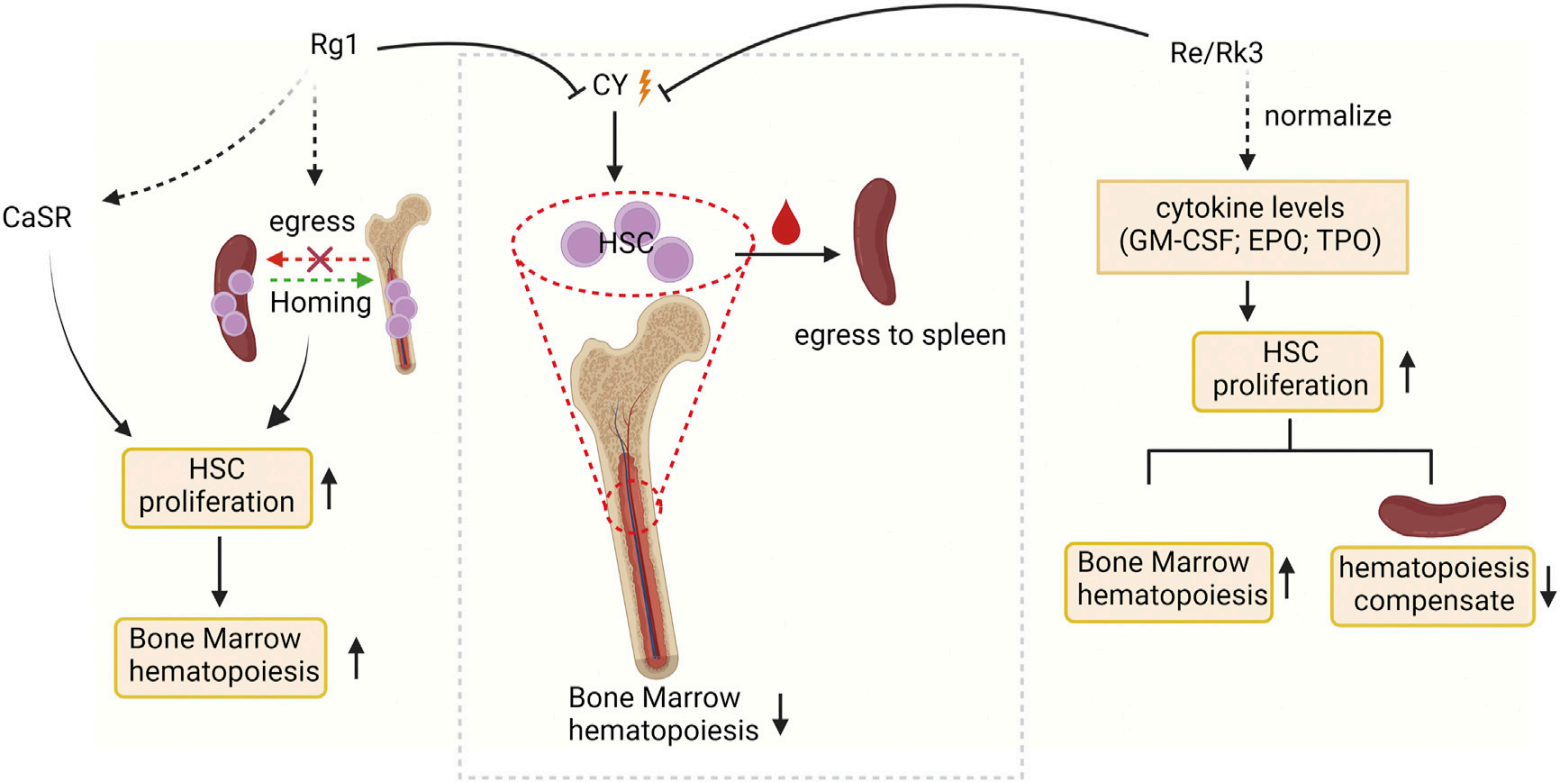
The targets of ginsenosides promoting HSC proliferation and stimulating bone marrow hematopoiesis at the molecular level. CY, cyclophosphamide; CaSR, Ca^2+^ sensitive receptor; GM-CSF, granulocyte-macrophage colony stimulatory factors; EPO, erythropoietin; and TPO, thrombopoietin.

External supplementation of HSCs is widely used to reconstruct damaged bone marrow (Ju et al., 2020). Bone marrow suppression and extramedullary hematopoiesis are often caused by the side effects of chemotherapy drugs (such as cyclophosphamide; CY) used by cancer patients, making stimulation of hematopoiesis, a critical issue in the context of cancer therapy in clinical practice (Wang et al., 2009; Ahlmann and Hempel, 2016; Hou et al., 2017). Ginsenosides relieve CY-induced myelosuppression by activating HSC proliferation (Figure 2). HSCs expansion in the bone marrow is strictly regulated by the HSCs niche (Pinho and Frenette, 2019). Multiple signal molecules are involved in HSCs-niche interactions, such as Ca^2+^ sensitive receptor (CaSR), and three cytokines, including granulocyte-macrophage colony stimulatory factors (GM-CSF), erythropoietin (EPO) and thrombopoietin (TPO), are essential for HSC proliferation (Szade et al., 2018). CaSR has been demonstrated that regulates calcium ion levels to maintain calcium homeostasis and plays critical regulatory roles in the retention and colonization of HSCs after transplantation (Cho et al., 2020; Uslu et al., 2020). Such the CaSR can be activated by ginsenoside Rg1 to relieve CY-induced inhibition of the proliferation of Lin-Sca-1+c-Kit + HSCs and CD3^+^ in mouse bone marrow and peripheral blood, restoring bone marrow function (Xu et al., 2012). Another study found that ginsenosides Re and Rk3 compensated hematopoietic function by increasing the secretion of cytokines (GM-CSF, TPO, EPO) to restore HSC proliferation. (Han et al., 2019). In addition, the compensatory hematopoiesis of the spleen is the key means to restore the normal hematopoietic function of the bone marrow. Ginsenoside Rg1 treatment of CY-induced myelosuppressive mice can improve bone marrow hematopoietic activity by improving the spleen niche and promoting the proliferation and homing of c-Kit + HSCs in the spleen (Liu HH. et al., 2015). As mentioned above, the niche may be an important target for ginsenosides to regulate HSC proliferation and assist recovery from myelosuppression. It is necessary to focus on whether the effects of ginsenosides on other cellular components in the stem cell niche feed back to the stem cells so that we can more fully understand the regulatory mechanisms of ginsenosides on stem cell fate specification.

#### 3.3.2 Effect on differentiation and self-renewal of HSCs in the aging process

**FIGURE 3:**
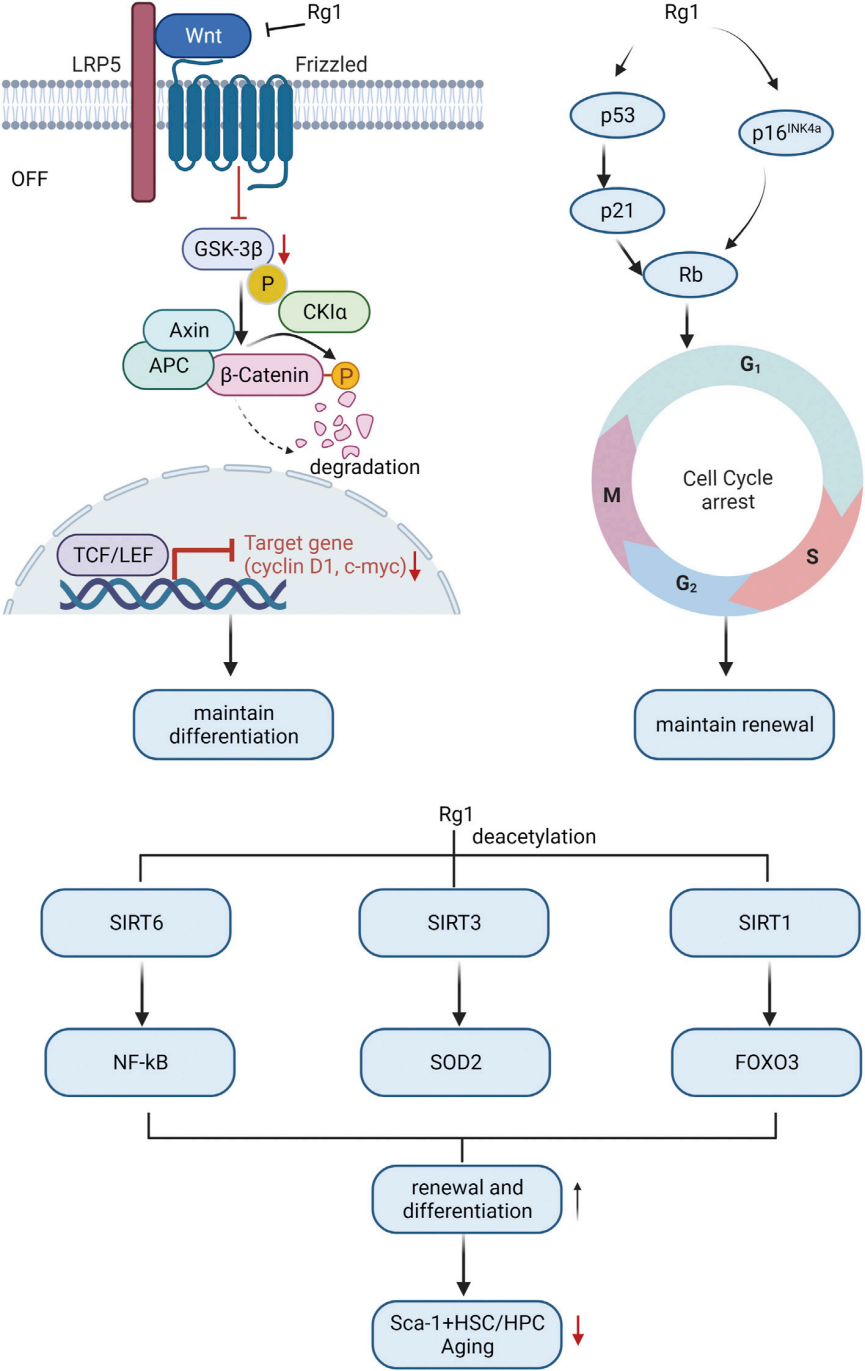
Molecular mechanism of ginsenosides affecting HSC differentiation and self-renewal ability to resist HSC aging.

Research indicates that HSC aging is linked to body aging since it impairs the self-renewal and differentiation ability of stem cells, resulting in decreased hematopoietic and immune function, and ultimately leading to tissue and organ structure and function deterioration throughout the body (Han et al., 2019). Therefore, it is meaningful to study the mechanism of HSC aging to elucidate the aging mechanism of the body. Ginsenoside Rg1 resisted HSC senescence to restore self-renewal and multi-differentiation abilities (Figure 3). Excessive ROS generated by oxidative stress may be the main mediator of stem cell senescence induced by excessive activation of Wnt/β-catenin signaling pathway (Zhang DY. et al., 2013). The proto-oncogene c-myc and cyclin D1 are target genes downstream of the Wnt pathway, and their overexpression may cause DNA damage and induce oxidative stress senescence (Shang et al., 2023). Ginsenoside Rg1 acts as a Wnt/β-catenin signal transduction inhibitor to inhibit Wnt target genes (such as cyclin D1 and c-myc) regulated by TCF/LEF transcription factors, thereby delaying LiCl and D-galactose-induced Sca-1 + HSC/HPC oxidative damage cascades restore their differentiation characteristics (Li et al., 2016; Wang et al., 2022a).

Cell cycle arrest is another main cause of HSC aging (Mohrin et al., 2015). Ginsenoside Rg1 antagonizes lead acetate, t-BHP, radiation, and d-galactose by repressing some key genes in the cell cycle regulator signaling pathway (p53-p21-Rb, p16^INK4a-Rb^, and p53-p21^Cip/Waf1^)-induced HSC senescence, improving HSC self-renewal capacity (Chen et al., 2014; Yue et al., 2014; Li et al., 2016; Cai et al., 2018).

Sirtuins alter protein activity and stability through lysine deacetylation is another important factor in regulating the cellular aging process (Lasigliè, 2021). The sirtuins SIRT1, SIRT3, and SIRT6 are key regulators of HSC lifespan (Wang et al., 2016; Fang et al., 2020; Wang et al., 2022b). Activation of SIRT6 by ginsenoside Rg1 inhibits NF-κB through H3K9 deacetylation, slows down t-BHP-induced Sca-1+HSC/HPC senescence, and enhances self-renewal and multi-differentiation abilities (Tang et al., 2015). Ginsenoside Rg1 activates SIRT3 to trigger deacetylation to enhance SOD2 activity and accelerate ROS clearance, slow down D-gal-induced Sca-1^+^HSC/HPC senescence, and promote HSC self-renewal and multi-differentiation ability (Zhou Y. et al., 2020). Ginsenoside Rg1-mediated SIRT1-FOXO3 promotes Sca-1^+^HSC/HPC multi-differentiation and self-renewal (Tang et al., 2020b), and inhibits gamma-ray-induced Sca-1^+^HSC/HPC senescence, which is dependent on deactivation of SIRT1/SIRT3 Acetylation (Tang et al., 2020a).

Overall, the mechanisms of ginsenoside in reducing HSCs aging mainly involve Wnt/β-catenin, cell cycle, and sirtuins-mediated senescence signaling pathway.

### 3.4 Cancer stem cells

CSCs exhibit qualities of stem cells and cancer cells, contributing to tumor growth, metastasis formation, and recurrence (Tanabe, 2022). CSCs initiate and maintain cancer initiation and progression based on stemness characteristics, namely, self-renewal and abnormal proliferation/differentiation (Eun et al., 2017).

#### 3.4.1 Effect on proliferation and self-renewal of CSCs

Wnt/β-catenin signaling is the main signaling pathway that promotes cancer cell stemness (Katoh and Katoh, 2022). Ginsenoside Rh2 inhibits cutaneous squamous cell carcinoma (SCC) proliferation by reducing the number of Wnt target gene Lgr5+ cells by inhibiting β-catenin signaling (Liu S. et al., 2015). Ginsenoside Rg3 and Rh2 reduced the self-renewal capacity of glioblastoma stem cells (GSC) by inhibiting the expression of transcription factor LCF1 and downstream Wnt target genes (c-myc, CCND1) of Wnt/β-catenin signaling (Ham et al., 2019). Notably, the anti-CSC capacity of Rh2 is better than that of ginsenoside Rg3, which supports that ginsenoside metabolites with fewer sugar groups have stronger anticancer activity.

Accumulating evidence indicates that EMT activation is abnormally high in CSCs, and there is a strong correlation between CSC stemness and EMT regulation (Tanabe et al., 2020). Recently, many studies have reported that ginsenosides exert anticancer effects by inhibiting EMT (Dai et al., 2019; Cai et al., 2021; Li et al., 2021). Ginsenoside Rg3R has been shown to reduce self-renewal in colorectal cancer cells (CRC) by targeting the SNAIL signaling pathway and modulating EMT features (Phi et al., 2019b). Ginsenoside Rk1/Rg5 inhibited EMT and self-renewal ability of A549 cells and reduced A549 stemness, which was dependent on the inhibition of TGF-b1-mediated downstream signaling pathways, including Smad2/3, NF-κB, ERK1/2, p38 MAPK and JNK (Kim et al., 2021). In addition, the hypoxic niche is the main place to maintain the stemness characteristics of CSCs. Nur77 is highly expressed in the hypoxic niche in a mouse model of colon cancer. Ginsenoside CK, as a Nur77 ligand target, prevents the Nur77-Akt activation circuit and inhibits CSCs proliferation and stemness (Zhang M. et al., 2022).

In summary, the regulation of ginsenosides on CSC stemness (especially self-renewal ability) involves a variety of signaling pathways. The key to solving the problem of targeted therapy is to further study whether these pathways exist independently or interact, which will be the key of tumor therapy in the context of ginsenoside.

#### 3.4.2 Effect on proliferation and self-renewal of CSCs in the aging process

One subtype of CSC, leukemia stem cells (LSCs), is a crucial origin of leukemia due to its high proliferation and abnormal growth (Chavez-Gonzalez et al., 2017). Inducing LSCs aging can reduce the number of LSCs, thus reducing the incidence of leukemia (Liu W. et al., 2022). Ginsenosides mainly induce the aging of LSCs by the following signals, slowing down or inhibiting the development of leukemia.

One of the mechanisms by which ginsenosides induce senescence in LSCs is their inhibitory potential for proliferation and self-renewal, involving deacetylation mediated by SIRT1 (one of the sirtuins members). Ginsenoside Rg1 downregulates the expression of SIRT1/TSC2 in CD34^+^CD38-LSCs, significantly increases the level of senescence marker SA-β-Gal, and reduces the unit of mixed colony-forming (a marker of proliferation ability), and reduces cell renewal and proliferation ability to induce LSCs senescence (Tang et al., 2020a).

Telomere is another pathway that ginsenosides participate in regulating the proliferation and self-renewal of LSCs to induce senescence. LSCs have relatively short telomeres, but they show higher levels of telomerase activity compared with normal cells. This enhanced telomerase activity may be an adaptive mechanism aimed at maintaining the continuous replication of LSCs and promoting leukemia development (Kuo and Bhatia, 2014). A recent study found that ginsenoside Rg1 inhibited CD34^+^CD38-LSCs proliferative activity, increased expression of telomere damage effector p16^INK4a^, and decreased human telomerase reverse transcriptase (hTERT, catalytic subunit of telomerase) to induce its replicative senescence for the treatment of leukemia (Tang et al., 2021). The above findings suggest that ginsenoside Rg1 inhibits the ability of LSCs to self-renewal and proliferation, and its targeted therapy based on the intervention of LSCs senescence is a valuable direction for healing leukemia in the future.

Noteworthy, we observed that the effects of ginsenosides on CSC and normal stem cell fate are asymmetric (inhibit CSC self-renewal, promote normal stem cell proliferation/differentiation), which may have multiple reasons. First, CSCs of various origins overexpress the glucose transporter GLUT1 (Maliekal et al., 2022). Due to their steroidal structure, ginsenosides have the property of recognizing GLUT carriers on tumor cell membranes (Chen et al., 2022). Ginsenoside Rh2 inhibits GLUT1-mediated aerobic glycolysis in tumor cells, and its role as a tumor energy blocker may be responsible for the inhibition of CSC self-renewal (Liu X Y et al., 2020). Second, ginsenoside Rb1 and its deglycosylated product compound K also decreased the expression of drug efflux pumps (ABCG2 and P-glycoprotein), inhibiting the resistance of CSCs to chemotherapeutic drugs (Deng et al., 2017). In addition, ginsenosides, as signal transduction regulators of the Wnt/β-catenin pathway, participate in the regulation of stem cell fate specification, with opposite effects on CSCs and normal stem cells. The regulation of ginsenosides on Wnt/β-catenin is mainly through regulating the activity of the intermediate GSK-3β to mediate the signal transmission triggered by β-catenin degradation/nuclear translocation, including the inhibition/activation of downstream transcription factors (TCF/LEF) and Wnt target genes (CCND1, c-myc, Lgr5), thereby affecting the function and fate of stem cells (Sferrazza et al., 2020). We speculate that ginsenosides inhibit CSC and stimulate normal stem cell proliferation/differentiation may be due to the different functions of Wnt target genes in CSC and normal stem cells. However, further verification *in vivo* or clinical experiments is needed to support this point of view. In conclusion, ginsenosides show potential as anticancer drugs, but further research and clinical trials are currently needed to determine their efficacy and safety.

### 3.5 Other kinds of adult stem cells

Although the effects of ginsenosides on other types of adult stem cells are not well understood, existing findings suggest a potential multifunctional regulatory capacity. For example, Ginsenoside Rd can stimulate the proliferation of intestinal stem cells (marked by Bmi and Msi-1) in rat inflammatory bowel disease model and promote the differentiation into intestinal epithelial cells expressing CDX-2, thereby restoring intestinal function (Yang et al., 2020). Ginsenoside Rg1 promotes the odontogenic differentiation of human dental pulp stem cells (hDPSCs) by upregulating osteogenesis-promoting factors, including bone morphogenetic protein-2 (BMP-2) and fibroblast growth factor 2 (FGF2) (Wang et al., 2014). In addition, PPT induced the proliferation and differentiation of sperm stem cells and resisted busulfan-induced reproductive toxicity in male mice (Ji et al., 2007). These findings suggest that ginsenosides have a wide range of effects, contributing to tissue repair and regeneration, and holding potential for the treatment of various diseases. Strengthening the study of the molecular mechanism of ginsenosides on other types of adult stem cells will help its clinical application.

## 4 Conclusion

This review gives an overview of the fate specification effects of ginsenosides on adult stem cells from the perspective of physiology (including aging states) and pathology. However, currently most of studies we reviewed are based on *in vitro* model and we still lack knowledge of how ginsenosides affect stem cell proliferation and differentiation or relieve dysfunctions in those processes *in vivo* and whether ginsenosides have considerable value in clinical practice. To figure out such limitations and the great potential of ginsenosides in tissue repair, cell replacement and disease treatment, more research (especially *in vivo* studies in distinct species) should be done to unravel the underlying mechanism of ginsenosides in proliferation, differentiation and self-renewal of different stem cell types. Considering the continuous proceeding of research on ginsenosides and proliferation as well as differentiation processes in various stem cells, we believe the promise of ginsenosides in regenerative medicine and healthy aging will be attested soon.
